# Trust and Credibility in Web-Based Health Information: A Review and Agenda for Future Research

**DOI:** 10.2196/jmir.7579

**Published:** 2017-06-19

**Authors:** Laura Sbaffi, Jennifer Rowley

**Affiliations:** ^1^ Information School Department of Social Sciences University of Sheffield Sheffield United Kingdom; ^2^ Information Interaction Research Group Department of Languages, Information and Communication Manchester Metropolitan University Manchester United Kingdom

**Keywords:** literature review, trust, health information, information retrieval, web

## Abstract

**Background:**

Internet sources are becoming increasingly important in seeking health information, such that they may have a significant effect on health care decisions and outcomes. Hence, given the wide range of different sources of Web-based health information (WHI) from different organizations and individuals, it is important to understand how information seekers evaluate and select the sources that they use, and more specifically, how they assess their credibility and trustworthiness.

**Objective:**

The aim of this study was to review empirical studies on trust and credibility in the use of WHI. The article seeks to present a profile of the research conducted on trust and credibility in WHI seeking, to identify the factors that impact judgments of trustworthiness and credibility, and to explore the role of demographic factors affecting trust formation. On this basis, it aimed to identify the gaps in current knowledge and to propose an agenda for future research.

**Methods:**

A systematic literature review was conducted. Searches were conducted using a variety of combinations of the terms WHI, trust, credibility, and their variants in four multi-disciplinary and four health-oriented databases. Articles selected were published in English from 2000 onwards; this process generated 3827 unique records. After the application of the exclusion criteria, 73 were analyzed fully.

**Results:**

Interest in this topic has persisted over the last 15 years, with articles being published in medicine, social science, and computer science and originating mostly from the United States and the United Kingdom. Documents in the final dataset fell into 3 categories: (1) those using trust or credibility as a dependent variable, (2) those using trust or credibility as an independent variable, and (3) studies of the demographic factors that influence the role of trust or credibility in WHI seeking. There is a consensus that website design, clear layout, interactive features, and the authority of the owner have a positive effect on trust or credibility, whereas advertising has a negative effect. With regard to content features, authority of the author, ease of use, and content have a positive effect on trust or credibility formation. Demographic factors influencing trust formation are age, gender, and perceived health status.

**Conclusions:**

There is considerable scope for further research. This includes increased clarity of the interaction between the variables associated with health information seeking, increased consistency on the measurement of trust and credibility, a greater focus on specific WHI sources, and enhanced understanding of the impact of demographic variables on trust and credibility judgments.

## Introduction

People are increasingly seeking health information and advice online. Statistics from the Pew Research Center show that one in three adults in the United States go online to try to identify a diagnosis or to know more about a health complaint [1]. For the United Kingdom, a report from Oxford Internet Surveys indicates that the number of people going online to seek health information has doubled since 2005, from 37% to 69% [2]. Hence, the Internet is an important source of health information and advice, and the information obtained may have a significant effect on health care decisions and outcomes [3,4] and reduce anxiety and depression while increasing feelings of self-efficacy and empowerment [5-7]. Powell et al [5] suggest that online information is used to educate, reassure, and to sometimes challenge information received from health professionals. However, health information seekers encounter a plethora of Web-based and other sources of health information from a variety of organizations and individuals, and of varying quality, accuracy, and reliability [8-10]. This presents individuals with significant challenges in evaluating and selecting the sources to use, and more specifically, in assessing the credibility and trustworthiness of those sources [11-14]. Yet, in health information seeking, source evaluation is especially important because the information or advice gleaned may have a significant effect on health-related behavior and decisions [10,15]. Furthermore, research suggests that “meagre information evaluation skills” or low health literacy enhance consumers’ vulnerability [16,17], and that individuals with higher eHealth literacy gain more positive outcomes from health information searching including improved self-management of health care needs and more effective interactions with their physician [18]. In addition, Stvilia et al [19] found that consumers may lack the motivation or literacy skills to evaluate the information quality of health Web pages, and Chenet et al [20] suggest that digital inequalities may influence the extent of an individual’s health information repertoires. Hence, research that enhances understanding of the factors that influence the evaluation and selection processes associated with digital health information is important, and can inform the design of information literacy programs, health information content, health information systems, and the design of the interaction between patients and health care professionals.

Given the importance of the evaluation of WHI, and more specifically the role of trust and credibility judgments, there is a growing body of research in this area, and therefore, a continuing need to develop coherent reviews of the field as a basis for further research and to inform practice. Hence, this article undertakes a systematic literature review of the research that features the concepts of trust and credibility in WHI seeking. More specifically, it seeks to answer the following research questions:

RQ1: What is the profile of the research conducted on trust and credibility in WHI seeking?

RQ2: Which factors have been identified as impacting on judgments of trustworthiness and credibility in WHI seeking?

RQ3: Which factors, alongside trust and credibility, have been identified as influencing WHI seeking?

RQ4: What demographic factors affect trust formation in WHI seeking?

Given the importance of the trustworthiness of the health information gathered from digital sources, other authors have conducted literature reviews on this and related topics. Most of these were conducted a few years ago and, while retaining significant reference value, require updating [16,21-25]. There are also more recent reviews that focus on specific aspects of WHI assessment [17,26-29].

Another unique and important aspect of this review is its scope, in that it embraces both trust and credibility. Most prior reviews, and indeed much of the research, distinguishes between trust and credibility. Furthermore, for some authors, trust is defined as an antecedent to credibility (eg, [30-32]), but by other authors (eg, [6,25,33]) trust is viewed as the end result of a process in which credibility is only one of many components. To further add to the ambiguity, other authors regard trust and credibility as interchangeable (eg, [34]), or believe that trustworthiness is one of only two primary dimensions of credibility [26]. Hence, given the interweaving of the concepts of credibility and trust, it is appropriate to include research on both of these aspects in this review.

As suggested above, there are many definitions of trust and credibility in literature. This section provides some examples to orientate the reader and give context to this research. Tseng and Fogg [35] have argued that trust and credibility should not be used interchangeably, nor be considered synonyms; according to these authors, trust “indicates a positive belief about the perceived reliability of, dependability of, and confidence in a person, object or process” (p. 41). Rowley and Johnson [36] stated that trust is “a precursor to successful and effective adoption, interaction and ongoing commitment in the digital space” (p. 494). On the other hand, credibility can be defined as “a characteristic defined by reader judgments, (...) not necessarily equivalent to the actual quality of the information, such as its accuracy or truthfulness” ([37], p. 240). Self [38] regarded credibility as “believability, trust, perceived reliability, and dozens of other concepts and combinations” (p. 421). This paper considers “trust” and “credibility” as two aspects of the same concept, without entering the debate about their different nature [23,25,39,40] because the articles analyzed below use both terms without much discrimination, demonstrating that the issue of defining the true relationship between trust and credibility is still very much unresolved.

## Methods

A systematic literature review was carried out to highlight and explore the various aspects and applications of the concept of “trust” in digital health information. The review protocol selected was that proposed by Tranfield et al [41], which advocates an evidence-based approach (ie, the appraisal and synthesis of research evidence). The main advantage of a systematic literature review over a more traditional one (eg, overview or narrative) is the adoption of a “replicable, scientific and transparent process” ([41], p. 209). Key to Tranfield et al’s approach are three main stages, defined as planning, conducting, and reporting; this is also consistent with the guidelines proposed by the NHS Centre for Review and Dissemination [42]. The first stage involves the identification of the need for a study on a particular topic. In this study, prior research conducted by the authors in the area of trust formation in health-related Internet searches highlighted the spread and variety of trust and related concepts and uses in the academic literature, making it difficult, at times, to locate and select relevant and targeted research. Exploratory searches were conducted to identify the initial relevant search terms and strings. This process was then refined and reapplied throughout the entire search phase every time new search strings were recognized, in order to maximize coverage. The second stage (ie, the actual building of the dataset) involved the selection of suitable databases. Four multidisciplinary and four health-oriented databases were selected (Table 1). The search was conducted in the article title, abstract, and keywords fields.

**Table 1:** Review protocol: databases.

Type of database	Database name	Search fields	Number of final records	
Multidisciplinary	Scopus	Title and abstract and keywords	932	
	ScienceDirect	Title and abstract and keywords	117	
	Web of Science	Title and topic	787	
	ProQuest	All-except full text	1208	
Health-focus	Medline	Title and keywords	313	
	PubMed	Title and abstract	254	
	PsycINFO	Title and abstract	211	
	Cochrane Library	Title and abstract and keywords	5	
Total number of records				3827

An exhaustive series of search strings was employed in each database, accounting for synonyms, plurals, hyphenations, and multiple word combinations (eg, “information quality” or “quality of information,” and “ehealth” and its variants “e-Health” or “e-health”). Numerous combinations of words and strings were applied with Boolean operators “AND” and “OR” to broaden the search. Over 20 searches were conducted. Examples include:

[online health information] AND [trust]

[digital health information] AND [credibility]

[web health information] AND [information quality]

[health information] AND [trust] AND [online] OR [electronic]

**Figure 1:**
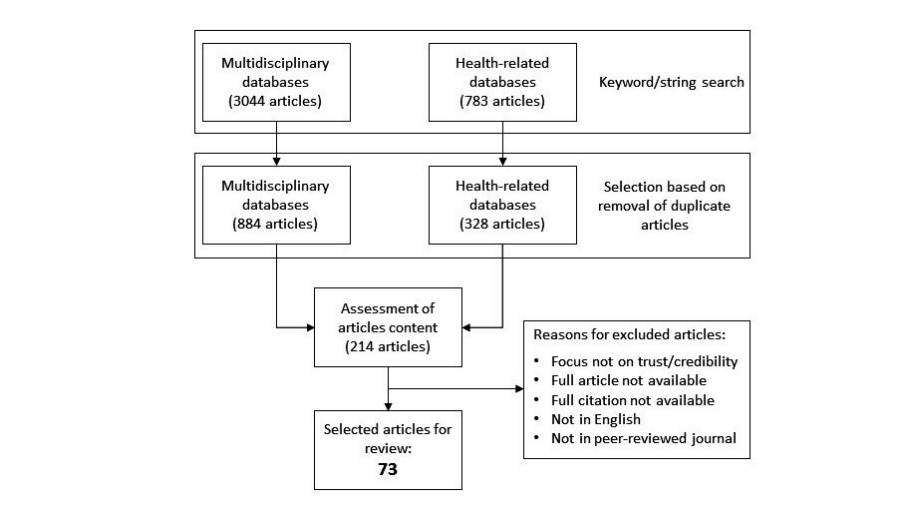
Search procedure for articles on trust and credibility in Web-based health information.

The search exercise, conducted in July 2016 on academic, peer-reviewed literature written in English from the year 2000 onward, identified a total of 3827 records (Table 1). All search results were exported to Microsoft Excel, collated, and all duplicates removed; this reduced the number of records to 1212 unique entries (Figure 1).

Next, an iterative process of refinement and exclusion was carried out on the records to optimize the emphasis on the proposed research topic. Each record (ie, titles, keywords, and abstracts) was scanned for relevance and source, and all articles off-topic, without a full citation, and written in languages other than English were discarded. All conference proceedings papers, books, and book chapters were also discarded, except for 2 conference papers that were retained because of their high citation rate ([43], 434 citations; [44], 180 citations). The final dataset comprised 73 journal articles that were downloaded and fully reviewed by the authors.

## Results

This section summarizes key aspects of the bibliographic profile of the literature (RQ1) before further elaboration on the topics covered by this research in response to the remaining research questions (RQ2, RQ3, and RQ4), which are discussed further below.

### RQ1: What Is the Profile of the Research Conducted on Trust and Credibility in Web-Based Health Information Seeking?

This section profiles the dataset in terms of the research methodologies used, and the distribution of articles over time, by discipline and by country.

#### Research Methodology

The majority of the titles (71 articles) in the final dataset were empirical studies largely conducted by means of quantitative research instruments (55 articles); nine studies were qualitative, seven used mixed-method approaches, and two were conceptual articles. The participants for the empirical studies were, in almost half of the cases (37/71 articles), adults between 18 and 65 years of age. Undergraduate students were the subject of the research in 20 articles and older people (usually 50-55 years and over) were studied in eight articles. Two studies [31,45] focused on both adults and undergraduate students, two more on high school students aged 11-19 years [12,13], one study was a comparative analysis between older (62+ years) and younger people (<26 years, nonstudents) [46], and one involved content analyses of websites, and therefore, did not require human participants [47].

In addition, there is no agreement on how trust and credibility are measured among different authors. For example, in 2007, Flanagin and Metzgen [31] adopted a 22-item scale to measure the credibility of health websites as a whole. In this scale, the authors included aspects like “colourful,” “aggressive,” “bold,” or “sophisticated” that have not been encountered in prior literature or reutilized since. In the same year, Sillence et al [48] adopted a 25-item scale to measure trust formation that included both information content and website design aspects. A few years later, Corritore et al [11] expanded on the concept of trust formation and acknowledged the complexity of the issue. These authors proposed a 34-item scale which encapsulated concepts of honesty, expertise, predictability, reputation, ease of use, and risk, but they did not include the visual or design aspect of the WHI experience. Recently, Johnson et al [49] attempted to merge previous measurement tools by creating a 55-item scale, including both design and content aspects of WHI. However, in contrast to other research [6,11,16,50], these authors did not explicitly include the concept of the risk associated with information seeking. Therefore, although progress has been made since 2000 in addressing and measuring trust (and credibility) formation, coherence and comprehensiveness are still to be achieved.

#### Discipline

**Figure 2:**
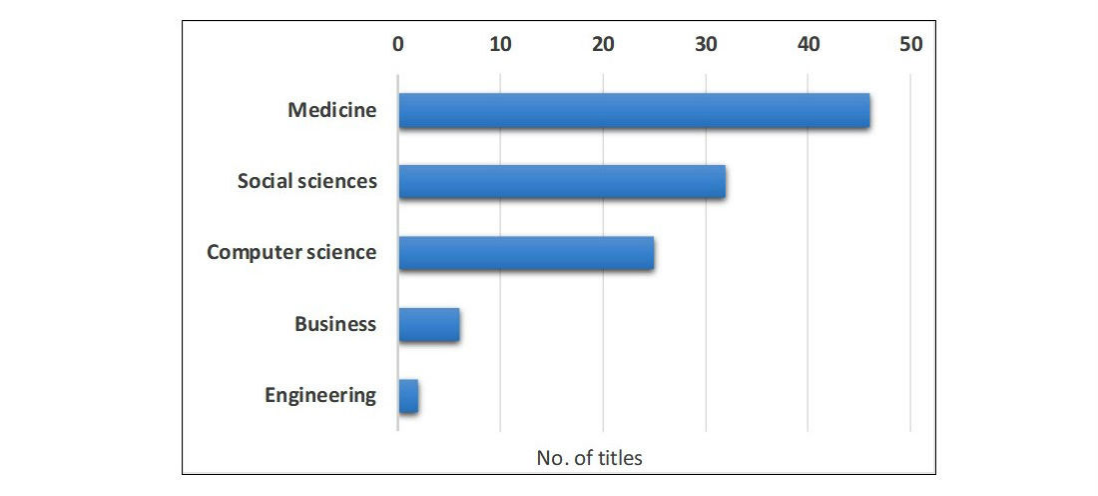
Article distribution by journal area of research. This chart has been informed by the Scopus-defined journal subject areas. The overall number of titles exceeds 73 because many journals have more than one subject area.

The final dataset was then categorized in terms of journal subject area, defined according to Scopus’ Scimago Journal and Country Rank (SJR) website (Figure 2). The main disciplines identified were medicine, accounting for more than half of all publications (46 articles), followed by social sciences (32 articles), and computer science (25 articles). The two most recurrent journal titles, with 8 articles each, were the Journal of Health Communication and the Journal of Medical Internet Research (medicine), followed by Social Science and Medicine (medicine or social sciences) and Decision Support Systems (business or computer science), with 3 articles.

#### Country

**Figure 3:**
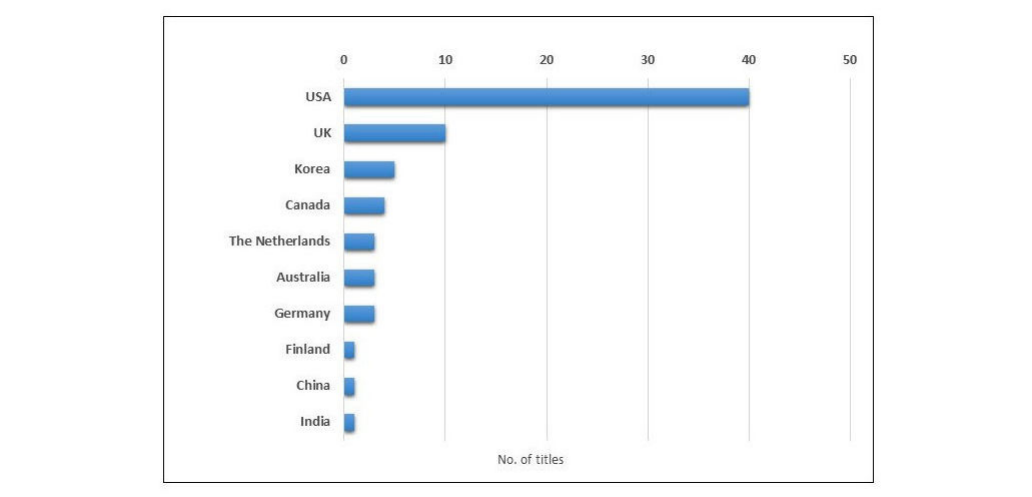
Article distribution by journal area of country.

All articles were also categorized on the basis of the country where the bulk of the research was carried out (Figure 3). he research conducted in the United Stated represents more than half of the entire dataset (40/73 articles, 54.8% of dataset). Research conducted in the United Kingdom followed in second place, but only accounting for 13.7% of the total dataset (10/73 articles). The other eight countries identified make up the remaining 27.4% (20/73 articles) of the dataset. In addition, three articles were the result of collaboration between two countries: Gray et al [12,13], between United Kingdom and United States, and Kitchens et al [10], between China and United States. Grouping the titles in terms of distribution by continent, the Americas account for 61.6% of the research (45/73 articles), Europe for 24.7% (18/73 articles), and Australasia for 13.7% (10/73 articles). This analysis highlights the wide gap in research output between the United States and the rest of the world in this area, indicating that there is still considerable scope for the study of trust in WHI before an exhaustive picture of the situation can be produced, particularly in those developing countries where access to technologies is less well established.

#### Timeline

**Figure 4:**
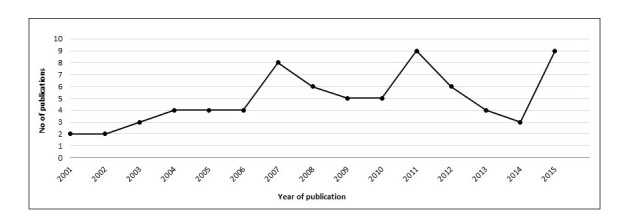
Publication of articles over time.

There is an established acknowledgment of the importance of research into trust and credibility in WHI seeking, and the number of publications has increased over the last 15 years, although slowly and with setbacks. The number of publications in the dataset never exceeded nine articles in any given year (Figure 4).

## Discussion

### RQ2: Which Factors Have Been Identified as Impacting on Judgments of Trustworthiness and Credibility in Web-Based Health Information Seeking?

The 34 articles in this category argue that trust (or credibility) can be defined as the end result of a series of judgments people apply during their online search processes. Such judgments are usually exercised on both the design features of websites and the content features of the information found. Tables 2 and 3 summarize the factors influencing trust formation. They are coded to show whether they have a positive or negative effect on trust formation, and whether the authors are using the terms “credibility” or “trust” in their research. In two instances [51,52], “Evaluation” (“E”) is used throughout, although the research is contextualized with reference to trust and credibility. In tables 2 and 3, factors are listed in order of decreasing number of citations.

There might be some overlap between the features identified as design and those as content, due to the personal interpretation that some authors provide of certain aspects. For example, discussion groups could be classed as a content feature, implying access to information created by other peers rather than professionals, and indeed, Sillence et al’s [44] discussion groups are classified as content features. However, in this review, the distinction is based on whether the website offers a link to a discussion forum or group to its users (design feature) or the material discussed in the forum or group is used for study purposes (content feature). In addition, personalization (here reported as design feature) could also encompass both design and content aspects as, for example, cookies could help remember preferences (design), and include opportunities to interact with other people on the site (content). However, in the articles analyzed here, only design aspects were discussed under the umbrella “personalization.”

When discussing design features, many authors agree that a clear layout of the website is a strong positive factor influencing trust formation; the presence of contact details and the authority of the owner of the website follow as the second and third most cited factors to consider when addressing trust or credibility issues. The availability of interactive features is another desirable trait of Web design, but it should be noted that the concept of “interactive” has been evolving with time. For example, Walther et al [53] defined a website as “dynamic” if it had changing features such as graphics and fonts. However, Diviani et al [52], more than 10 years later, considered “interactive features” the presence of chat-rooms and fora linked to the website. Interestingly, Fogg et al [43] were the only authors to take into consideration elements such as functionality, customer service, and affiliations.

Much less work has been conducted on negative aspects of design features (Table 2). There is consensus among authors that the presence of explicit advertising on a health website is the least desirable visual feature. Other than this, the majority of negative website design features discussed in the literature emerge from the work of Sillence et al conducted in early 2000s [6,44,48,54]. This is an interesting finding as, although the positive aspects can, in principle, be argued in opposite terms, very little explicit distinction is made between what is good and what is bad in WHI.

**Table 2:** Factors influencing trust judgments with trust or credibility as dependent variable—design features (C=credibility, T=trust, and E=evaluation).

Features	Impact	Factor	Articles using this factor
Website design features	Positive	Clear layout/design	[6] (T); [8] (C); [31] (C); [33] (T); [43] (C); [44] (T); [46] (C); [48] (T); [49] (T); [51] (E); [54] (T); [55] (T); [56] (C); [57] (T); [58] (T); [59] (T); [60] (C)
		Contact details	[8] (C); [30] (C); [32] (C); [34] (C & T); [52] (E); [61] (T); [62] (C)
		Authority of owner	[8] (C); [30] (C); [32] (C); [34] (C & T); [48] (T); [52] (E); [53] (C)
		Interactive features	[6] (T); [48] (T); [52] (E); [53] (C); [63] (T); [61] (T)
		Brand/logo	[33] (T); [49] (T); [54] (T); [55] (T); [61] (T)
		External links	[8] (C); [32] (C); [34] (C & T); [64] (C)
		Quality seal/endorsement	[8] (C); [30] (C); [52] (E); [58] (T)
		Navigation aids	[6] (T); [30] (C); [32] (C)
		Pictures	[6] (T); [32] (C); [52] (E)
		Discussion groups	[6] (T); [48] (T); [61] (T)
		Privacy policy	[30] (C); [32] (C)
		Identity of sponsor	[34] (C & T); [43] (C)
		Health on the Net (HON) network	[30] (C); [52] (E)
		Personalisation	[57] (T); [59] (T)
		Functionality	[43] (C)
		Customer service	[43] (C)
		Affiliations	[43] (C)
		Easy to access	[57] (T)
		FAQ section	[6] (T)
	Negative	Advertising	[6] (T); [8] (C); [30] (C); [43] (C); [44] (T); [52] (E); [53] (C); [54] (T); [64] (C); [58] (T)
		Slow	[6] (T); [44] (T); [48] (T); [54] (T)
		Complex layout/design	[6] (T); [31] (C); [44] (T)
		Boring layout/design	[6] (T); [31] (C); [44] (T)
		Inappropriate name	[6] (T); [31] (C); [44] (T)
		No navigation aids	[6] (T); [31] (C); [44] (T)
		No/poor search facility	[6] (T); [31] (C); [44] (T)
		Commercial domain	[53] (C); [65] (T)
		Uncaring/unconcerned	[66] (C)
		Textual deficit	[64] (C)

The most widely discussed positive aspect of content features in the literature (Table 3) is the authority of the author, representing the level of expertise of the person or persons writing the information; this is followed by the credibility or trustworthiness of the information (in articles where the main focus was either trust or credibility). For example, Freeman and Spyridakis [37] in their study on the measure of Web-based credibility, described credibility itself as being defined by two main components: trustworthiness and expertise, but Corritore et al [11] found that credibility was one of the direct predictors of trust. The objectivity of the information is also equally important and usually defined by authors in terms of how impartial and unbiased the source is. Ease of use, the fourth most common factor identified in the literature, is here seen as an aspect of content and how the information is made intelligible, and is based on a user’s perceptions [11].

**Table 3:** Factors influencing trust judgments with trust or credibility as dependent variable—content features (C=credibility, T=trust, and E=evaluation).

Feature	Impact	Factor	Articles using this factor
Information content features	Positive	Authority of author	[8] (C); [11] (T); [12] (C); [30] (C); [32] (C); [33] (T); [34] (C & T); [43] (C); [48] (T); [49] (T); [50] (T); [52] (E); [55] (T); [67] (T); [66] (C); [58] (T); [61] (T); [62] (C); [65] (T); [68] (C); [69] (T)
		Credibility/trustworthiness	[11] (T); [12] (C); [33] (T); [49] (T); [53] (C); [54] (T); [64] (C); [55] (T); [66] (C); [61] (T); [62] (C); [69] (T)
		Objectivity	[6] (T); [11] (T); [33] (T); [34] (C & T); [44] (T); [46] (C); [48] (T); [49] (T); [54] (T); [55] (T); [66] (C); [59] (T); [61] (T)
		Ease of use	[11] (T); [33] (T); [48] (T); [49] (T); [51] (E); [52] (E); [64] (C); [63] (T); [55] (T); [57] (T); [61] (T)
		Readability	[6] (T); [8] (C); [43] (C); [44] (T); [52] (E); [54] (T); [63] (T)
		Familiarity	[31] (C); [43] (C); [49] (T); [67] (T); [56] (C); [61] (T); [65] (T)
		Currency (up-to-date)	[8] (C); [30] (C); [34] (C & T); [52] (E); [70] (C); [58] (T)
		Triangulation	[33] (T); [34] (C & T); [49] (T); [55] (T); [58] (T); [65] (T)
		Usefulness	[33] (T); [43] (C); [49] (T); [63] (T); [55] (T); [71] (T)
		References	[30] (C); [52] (E); [70] (C); [65] (T)
		Relevance	[6] (T); [31] (C); [43] (C); [44] (T); [48] (T); [54] (T); [63] (T); [71] (T)
		Recommended by others	[33] (T); [49] (T); [55] (T)
		Accuracy	[43] (C); [52] (E); [71] (T)
		Quality	[50] (T); [57] (T); [59] (T)
		Clarity/understandability	[48] (T); [63] (T); [71] (T)
		Adequacy	[51] (E); [63] (T); [68] (C); [69] (T); [71] (T)
		Quotations	[30] (C); [70] (C)
		Comprehensiveness	[52] (E); [66] (C)
		Statistics	[30] (C); [70] (C)
		Empathy	[12] (C)
	Negative	Risk	[11] (T); [50] (T); [63] (T)
		Inappropriate information	[6] (T); [44] (T); [54] (T)
		Irrelevant information	[6] (T); [44] (T); [54] (T)
		Complex information	[52] (E)
		Bias of information	[43] (C)

Readability, familiarity, currency, triangulation, and usefulness are other factors that have fuelled researchers’ interest and which have been the subject of a number of studies. Less common positive content aspects, mentioned only marginally in literature, include the presence of quotations, statistics, and empathy.

The discussion around the negative aspects of the content characteristics of WHI has been much more limited and fragmented. Very few authors have dedicated time to assess what hinders trust (or credibility) in WHI sources. Some researchers have discussed the concept of risk being associated with trust, particularly from a philosophical perspective, stressing that every transaction that requires trust has a degree of associated risk [11,50,63]. The detrimental effects on trust formation deriving from the information being inappropriate or irrelevant, have only been discussed by Sillence and coauthors [6,44,54], with one work focusing on the complexity of the information [52] and another on the bias of the information [43].

### RQ3: Which Factors, Alongside Trust and Credibility, Have Been Identified as Influencing Web-Based Health Information Seeking?

Nine articles (Table 4) use trust as an independent variable in their research model and view it as an antecedent to (1) the evaluation of information “quality,” or (2) to the intention to “use” the information found. In addition, Escoffery et al [72] report on the ranking that college students give to a number of criteria when looking for WHI.

#### Information Quality

Bates at al [73] showed how trustworthiness, together with truthfulness, readability, and completeness can influence the quality of information; in addition, more readable health websites can improve the quality perception of the information, but this does not seem to have an effect on the overall trust [74]. Stvilia et al [19] highlighted how the quality of the information is informed by many aspects and trustworthiness is only one of them, together with accuracy, reliability, credibility, and clarity to mention only the most relevant. In a recent article, Kitchens et al [10] showed that the quality of health information is dependent on trust as well as accuracy, but also on the relevance of so-called “referral links” (ie, other websites that link to the main one).

**Table 4:** Factors, alongside trust or credibility (independent variable), influencing online health information seeking.

Outcome variable	Related article	Major findings
Quality of information	[10]	The quality of health information is dependent on information accuracy and trustworthiness. Quality is then linked to website importance via the number and importance of referral links (ie, links to the website and importance of those websites that link to it)
	[19]	The quality of information is informed by many factors; the first five, in decreasing order of importance, are: accuracy, reliability, credibility, trustworthiness and clarity
	[73]	Trustworthiness, truthfulness, readability and completeness are the main factors influencing the quality of information
	[74]	Making a health website more readable improves quality perception of the information, but there is no effect on trust
Use of the information	[75]	Trust, together with the importance given to written media, concerns for one’s own health, importance given to the opinion of HCPs and perceived usefulness, is an antecedent of the intention to use the information
	[76]	Perceived benefit, high interactivity and trust positively affect health information use, as well as satisfaction and long-term loyalty
	[77]	Trust, together with demographics, experience, salience of info and health beliefs, positively influences the intention to use
	[78]	Older people have concerns about the credibility of online health information and the less they trust it, the less they discuss it with their doctors
	[57]	Usability and usefulness contribute to trust formation which, in return, is key to return and reuse a source of information
Factual list	[72]	College students have ranked a series of criteria to consider when looking for online health information and accuracy, credibility and currency of the information are the top three

#### Intention to Use the Information

Five articles discuss the factors that affect intention to use health information found online. Lemire et al [75] proposed how trust is linked to the use of the information, but only in conjunction with the importance given to the opinion of health care professionals (HCPs) and the perceived usefulness of the information. Lee et al [76] argued that perceived personal benefit, highly interactive websites, and long-term loyalty to specific resources can, together with trust, affect health information use. In two more recent empirical studies, Sheng and Simpson [77] and Pannor Silver [78] discussed the issue of information use from the perspective of older people. Sheng and Simpson [77] claimed that some demographic factors can still influence health information seeking in senior users, particularly age, education, and income, but such factors only bear weight if considered in association with one’s own experience with the resources and health beliefs. Pannor Silver [78] highlighted a number of barriers that prevent aging people from trusting and, therefore, using digital health information, particularly in relation to poor e-literacy skills and lack of critical judgment of the quality of the information. Fisher et al [57] conducted a study in Australia on how usability and usefulness of medical websites are crucial to build trust in users and how such trust is then applied to return to and reuse a specific resource.

### RQ4: What Demographic Factors Affect Trust Formation in Web-Based Health Information Seeking?

Table 5 lists the 24 papers analyzing demographic aspects in connection with the concept of trust in WHI.

**Table 5:** Demographic factors influencing trust formation in Web-based health information seeking.

Factor	Hypothesis	Related articles
Gender	Women go online/trust online info more than men	[45,67,79-82]
	No difference between genders	[30,45,83-85]
Education	People with higher education levels go online/trust online info more	[45,79-81,83,85,86]
	No differences due to education level	[87]
Health status	People with poor health go online/trust online info more	[80,86,88]
	People with good health look for offline health info resources more	[80]
	People with good health go online/trust online info more	[79,83]
	Positive relationship between trust and self-efficacy belief in taking care of one’s health	[89,90]
	No differences due to health status	[85,87]
Income	People with higher income go online/trust online info more	[45,79-81,83,85,91]
	No differences due to income	[87]
Age	Younger people (25-55 years) go online/trust online info more	[45,79,81,85,86]
	Younger people (25-55 years) trust online info less than older people	[37]
	Older people (usually 55+) do not trust online info and prefer offline resources	[83,84,87,91]
	Articles discussing how young adults (from teenagers to college students) judge and trust online info	[12,13,33,34,49,55,60,71,72,92]
	Articles discussing how elderly people judge and trust online info	[58,77,78,84,93-96]
	Articles comparing young adults versus elderly online behaviour and trust	[46,97]
Health literacy	High health literacy and seeing HCPs often promote online trust	[12,13,98]
	No differences due to health literacy	[85]
Race	White people go online/trust online info more than black people	[81,86]
Parental status	Parents, regardless of gender, behave similarly online	[82]
High/low skilled Web users	The higher the skills the lower the trust in the info	[37,67,97]

Widely discussed in literature are the two extremes of the age spectrum, the young and the old, and their alleged profound differences in selecting, evaluating, and trusting WHI. Old age has been associated with an overall low trust in Web resources [83,84,87,91], as people in this age group rely more on interpersonal relations with physicians, pharmacists, friends, and family [84]; in this respect, the better the quality of doctor-patient communications or other health care providers, the less people tend to go online to look for alternative health resources [86]. Medlock et al [93] found that, indeed, older people depend more on face-to-face interactions with doctors (first) and pharmacists (second), but the use of the Internet is their third chosen source on health information, and aging people who use the Internet more than their peers tend to use all other health information sources as well. McMillan and Macias [94] made the distinction between “health technologists,” who are younger seniors using online resources frequently and reporting higher trust in the information retrieved and “health traditionalists,” who are the older segment of seniors using the Internet seldom and, therefore, trusting its information much less. Distrust in Web-based resources is associated with difficulties in navigating through large amounts of often confusing information. Zulman et al [95] noted that health websites reporting clearer features that identify easily the source and authorship of the information would promote the use of the Internet among more senior people. This age group acknowledges the importance of selecting trustworthy, credible information but lacks the experience in identifying what trust indicators should be used, hence, simpler and clearer layouts would be easier to navigate. The use of the Internet is associated with an intrinsic trust in the information found and with the perception that searches are easy to carry out, but such perception and the associated feeling of trust decrease with age [63]. A comparative study conducted in Germany by Feufel and Stahl [97] on young and elderly people, emphasized how highly skilled Internet users (identified as young, with high levels of education and more Internet experience) are more confident about the quality of the information retrieved, achieve more focused results, and conduct searches to objectively inform themselves as opposed to low skilled people (identified as older, with lower education levels and patchy Internet experience), who perform less effective searches only to confirm their own preexisting opinions on a topic. Better Internet skills have also been correlated with less trust in the health information [37].

Not surprisingly, at the opposite end of the age scale, young adults, in particular teenage users, seem to experience the same lack of judgmental skills of their much older peers when evaluating health information on the Web. In studies conducted on UK and US adolescents, Gray et al [12,13] reported that young people have difficulties in evaluating online information, which are further exacerbated by low functional, critical, and interactive literacy skills. Very young adults base their assessments of WHI on aesthetics prompts, how familiar they are with a certain website [92], and on how easy it is to access the information [55]. The confidence in their own search strategies contributes to increasing the trust in the information [92]. However, it might be that such confidence is entirely subjective and unjustified, particularly if other older and more expert people are asked to review the same sources [12,13]. Johnson et al [55] found that assessment skills become deeper and more content-oriented with age, so that university students in their third year of study show more discerning judgments than their peers in the first year.

The intermediate age group, here generally defined as comprising people between 25-55 years, shows an online behavior that is more influenced by other demographic factors, particularly education and income levels. As reported in Table 5, several studies have confirmed that people with higher incomes [79-81,83,85,91] and a higher education level [79-81,83,85,86] trust and use digital health information more than people in lower socioeconomic groups. A study conducted in Australia by Dart [45] showed how people from high socioeconomic and university-based backgrounds used the Internet for health information more than disadvantaged people but, in spite of considerable differences in the Internet use, neither group particularly trusted online information. Only Ye [87] reports conflicting findings; according to this research, neither personal capital (ie, income, age, education, and health status) nor social capital (one’s network of social interactions) have an impact on trust judgments of health digital information. A study by Dutta-Bergman [99] described how, at the dawn of the Internet era, trust in WHI was segmented according to the sources accessed; for example, younger people with strong health beliefs would trust local doctors’ websites the most, whereas less educated people with weaker health beliefs would trust in hospitals more; people with higher income and education would prefer medical universities’ websites for their information needs. A more recent trend, due to the widespread use of social media, is the willingness to share health information with others online.

The perception of one’s own health status is another determining factor in health information evaluation; however, research has identified conflicting trends. Ye [89] reported that trust in WHI is associated with the ability to assess and look after one’s own health (self-efficacy) and with negative emotions due to perceived poor health. This finding is in agreement with Atkinson et al [80] and Hou and Shim [86], who have also demonstrated how people in good health tend to use offline information sources more. This contrasts with findings from Cotten and Gupta [79] and Soederberg et al [107] who assert that a good health status is indicative of more online activity. Other authors [85,87] have shown that there is no relationship between perceived health status and trust in health information.

A considerable number of the studies reviewed cover the issue of gender, but mostly as part of a larger set of demographic attributes (see Table 5); some authors agree that women use and trust the Internet more than men when it comes to health problems [45,67,79-82]. However, a number of studies showed no differences [66,83-85], leaving the gender debate open and in need of further research. Only two studies focus on gender differences in online health searches behavior: one from Korea [67]and one from the United States [82]. In contrast, the role of gender in influencing online trust judgments, in contexts other than health, has received more attention (eg, [100,101]).

### The Remaining Themes

Four articles focused on information sources and trust or credibility. LaValley et al [102] reported that almost 3/4 of Americans use commercially sponsored websites to satisfy their health information requirements, but stressed how different website types have different reasons for sponsoring health information, which may affect the website’s content and design. Hu and Sundar [103] showed that websites are preferred to bulletin boards, home pages, and blogs, and credibility was strongly associated with users’ perceptions of the relevance of the message associated to a certain source. Stoerger [47] found that websites with lower credibility levels were associated with a lot of interactive features and advertising. In an earlier article by Rains [70], based on the Health Information National Trends Survey (HINTS), the author demonstrated a link between a person’s trust in mass media and one’s health care provider and an increased use in WHI resources.

Two articles discussed trust from a theoretical perspective. Sillence and Briggs [104] explained how ubiquitous computing [105] has long-term and still unknown implications for the health care sector because it produces a shift of people’s trust from physicians to artificial agents (ie, computers). Singal and Shruti [106] claimed that there is no standard that exemplifies how to make trust decisions in health; however the authors envisaged to develop a technique to rerank search results using trust as a determining factor so that the more trustworthy a website, the higher its position in a result list.

### Toward a Future Research Agenda

This article reports on a systematic literature review of the peer reviewed literature exploring the concept of trust and credibility formation in WHI seeking. The review demonstrates that there is still no consensus of the relationship between the terms “trust” and “credibility”. This review also demonstrates that trust and credibility have been investigated both as the dependent variable, representing the end product of a series of cues and factors influencing the process of information seeking and as independent variable, alongside other variables associated with the quality or use of information. In addition, other research has examined the impact of demographic variables such as gender and age, on trust and credibility judgments in WHI seeking. Nevertheless, while there is a growing body of research in this area, given the importance of the trustworthiness of WHI, there is considerable scope for future research and theoretical development in this area. This includes:

Conceptual or theoretical: It would be of considerable benefit to be able to arrive at a consensus on the relationships between the various variables associated with research into WHI seeking. For example, there needs to be further consideration as to whether trust and credibility or information quality are the most important outcome variables. We propose that consideration of the context may be important in differentiating between trust and credibility, with, for example, trust being the appropriate term to use when information is used to inform a decision or action. This stance would also necessitate the development of an improved understanding of the relationship between trust and risk in digital health information seeking. Having established, or at least, further explored the relationships between trust and credibility, it will be important to increase understanding of the key influencing factors, and the extent to which context might impact on these.

Methods and measurement: Most research on trust and credibility in WHI seeking has adopted a quantitative approach. The purpose of quantitative studies is typically to test theory. However, in the absence of a consensus on definitions of variables and the dominant relationships between them, such studies are unlikely to lead to an integrated and coherent body of knowledge. This is further undermined by the considerable variability in the measurement scales used for trust and credibility and related variables. This needs to be addressed by a much greater number of qualitative studies that offer deeper insights into the context, processes, and judgments associated with WHI seeking and the relationship between these.

Topics: Most studies have investigated the factors that influence trust judgment in relation to WHI in general. Hence, there is scope for more studies that take into consideration judgments on specific health information sources, including specific websites and social media platforms, and the role of the owner and community associated with these platforms in influencing trust judgments. This review has not embraced research on trust regarding health information received in social support groups; this would also be an important agenda for future research. In addition, most prior research has privileged factors that have a positive effect on trust judgments, with few reporting on those factors (such as advertising) that might undermine trust. Finally, the dynamic between trust and risk deserves greater attention, particularly with regard to patients’ perceptions of the seriousness of their complaint.

Impact of demographic variables: There is evidence that various demographic variables (eg, age, income, and gender) may influence WHI-seeking behaviors, but the evidence that this also impacts on their trust judgments is scant. Further research is needed in this area. In particular, the research on disadvantaged groups has focused on identifying their needs, but little work has been done on how these needs and the ability of members of these groups to discriminate between trustworthy and untrustworthy information can be enhanced. In addition, whereas some research has been conducted on the role of gender on trust formation in information seeking, which makes links to the role of the psycho-social context, there is considerable scope for further research into the role of this context in trust formation in health information seeking. Finally, the current research base focuses on health information seekers in the United States, United Kingdom, and Australia. There is a need for research in countries where not only technological differences can play a role in information seeking, but also culture and, more specifically, trust formation and relationships with health organizations and professionals are likely to differ from non-western countries.

## Abbreviations

HCP: health care professional
WHI: Web-based health information
